# Genuine Charity

**Published:** 1875-04

**Authors:** 


### GENUINE CHARITY.

About a century ago, John Howard was a common English gentleman. On his estates was a village which had been subject for generations to fevers. The tenants of its miserable hovels had always dwindled and pined away, from the effects of the pestilent exhalations from the neighboring swamps and fens. This benevolent, simple-minded gentleman, thought he would improve the health and condition of these poor people. He accordingly went to work, drained the ground, rebuilt the cottages, reformed the mode of living, and, in a little while, had a model village, full of health, and happiness, and beauty. This was repeated, and this method of reform spread. Shortly afterward Mr. Howard was chosen Sheriff of Bedfordshire. He went into Bedford Jail, once the home of John Bunyan. There he found the poor prisoners suffering cruelly. He pursued his work of benevolence. He asked for statistics. The authorities demanded precedents for such proceedings; but John Howard pressed on, and just one hundred years ago you might have seen him travelling all through England, and going into every jail. Then out came the statistics of pauperism and crime. Howard crossed the channel, spreading his glorious work in Europe. This is only one case. What have we here? We have no hard State laws, but Christian charity, infusing itself into legislation; and when we have noble Christian men and women organized into societies, may we not almost look for miracles from such a touch? To give out-door relief is wise action. The man who is able to work and will not, ought to die. Let us look out for work for poor people, and send the little children out into the country where they may learn to live by honest labor.

---

The house should bear witness in all its economy that human culture is the end to which it is built and garnished. It stands there under the sun and moon, to ends analogous and not less noble than theirs.

---

We should creep before we walk, walk before we run, and run before we ride.

